# Medical News and Miscellany

**Published:** 1896-03

**Authors:** 


### Medical News and Miscellany.

**Dr. Daniel Lewis** has resigned the editorship of the *Medical Review.*

**Dr. C. A. Smith,** of Tyler, Chief Surgeon of the "Cotton Belt" Railroad, in renewing his subscription, says: "*Long live the Red Back.*"

The *Western Reserve Medical Journal*, of Cleveland, Ohio, has been retired; and is succeeded by the *Cleveland Journal of Medicine.* P. Max Foshey, M. D., is managing editor; Dr. H. S. Upson is editor.

**Dr. Wm. R. Pfeuffer,** of New Braunfels, a graduate of the College of Physicians and Surgeons of New York, class of '95, has located at Rockdale, Texas.

---

**Dr. Joseph Jones,** Surgeon-General of the United Confederate Veteran Association, the well known author and teacher, died at his home, in New Orleans, on the 17th of February, ult.

---

**Dr. McKay** "pulled off" that "World's Congress" on schedule time;—but from all accounts it was pulled green,—premature. It reminds one of the two tailors in Thread-needle-street, who resolved—"We are the people."—World's Congress;—yes: very.

---

**The Standing Chairman.**—A certain member of the Texas State Medical Association has been appointed chairman of a certain section half a dozen times,—is now chairman of same section: but the oldest inhabitant has no recollection of his ever having made a report or done anything whatever in connection with the section.

---

**A case** of intestinal obstruction, necessitating laparotomy, has been reported, caused by the impaction of a lot of salol tablets which the patient had been taking. It should be borne in mind that salol is insoluble, or nearly so, and its administration in compressed form is attended with danger, as it would appear from the above.

---

**Dr. Geo. Dock,** of the University of Michigan, has declined the chair of Pathology and Bacteriology in Jefferson Medical College, to which he was recently elected. We are surprised to hear this, inasmuch as we had thought it a distinguished honor which his *alma mater* proposed to confer upon him, and that he would promptly accept it.

---

**Apropos:**—Comes now another alleged discovery of a cure for leprosy; this time, from the United States of Columbia. The *Medical Record* says that the government of Columbia has ordered that the lepers throughout that republic shall be treated by the new method discovered by Dr. Carsaquila, of Bogota; but no particulars are given. We suppose it is a *serum*, of course.

---

**"Blocks of Three."**—To induce prompt renewals and new subscriptions, the JOURNAL makes this offer for 1896: The subscription price is $2.00 a year. We will send three copies one year for $4.00, all new subscribers, or one renewal and two new names. That is, any one, subscriber or not, who will send us two new subscribers and $4.00, will receive the JOURNAL one year *free* of charge.

---

**Seasickness.**—Dr. A. D. Rockwall (*in New York Med. Record*) says that bromide of sodium, in about 30 grain doses, taken several times a day, will prevent seasickness. The treatment should be begun several days before embarking, kept up during the voyage, and continued a day or so after landing. It must be given to its full physiological effects; until "brominism,' is produced.

---

**Wanted.**—To complete files, the following back numbers of the "Red Back." Any one who will kindly send them, one or more or all, will be credited with same on subscription account, besides it will greatly oblige yours truly: Vol. 8—August '92, October '92, February '93, and March '93; Vol. 9—November and December, '93; Vol. 10—October '94 and June '95.

F. E. DANIEL.

---

**Still Another Want Supplied.**—The *Clinical Recorder*, W. S. Gottheil, M. D., editor; G. A. Sykes, publisher; 66 World Building, New York, has been started. Dr. Gottheil makes no apology for it, but says: "Our purpose is merely to furnish a practical record of clinical events, which shall be useful to the general practitioner and interesting to the specialist." Vol. 1, No. 1, received. We have placed the *Recorder* on our list, with pleasure.

---

**Prospective Publication.**—The genial and versatile Dr. G. Frank Lydston has in preparation a book of Doctor's stories, called "The Tales of a Talkative Doctor." The contents will comprise: "Character sketches from real life; psychological studies; satire of current medical follies; dialect stories and every day medical philosophy gleaned from the life of a busy doctor, together with the "rhodomontades of a sociable skull.'' We are sure it will be good. PROGRESS.

---

**Dr. Kitasato.**—The Japanese mail, says the *Medical Record*, brings, by way of Vancouver, the report of a great excitement in medical circles, due to the announcement by Dr. Kitasato, that he has produced a serum capable of arresting the plague, curing diphtheria, arresting cholera, and neutralizing the effect of the bacillus lepræ. The discoverer's great reputation gives much weight to the claim among Japan's physicians.

"By my troth he doth protest *too much.*"

---

**Attention** is called to the action of the Terrell Medical Association, published elsewhere. It is not after the stereotyped form of "Society Proceedings," giving "cases," but upon a great and important movement, which is worthy of and demands the cooperation of all good medical men.

---

**The American Medical Publishers' Association** will hold its third annual meeting in Atlanta, Ga., Monday, May 4th, and considering the many recent applications for membership, a large attendance is assured. A number of new and important topics have been suggested for discussion, and the programme will include papers from experienced publishers. Members and others desiring to contribute papers will be furnished valuable information upon communicating with the secretary, Charles Wood Fassett, St. Joseph, Mo.

---

The JOURNAL is in receipt of a brief note from Dr. Merriman. He proposes to "drop in" again shortly, and writes now, he says, only to inquire if the X Rays is going to enable an impecunious subscriber, for instance, to raise an X? And speaking of "X's," he says his wife rather suspects that "Dan'els" must have heard of that $10 fee he received last year, else he would not call him the "fat fee" losopher; she says it looks suspicious. By the bye, Dr. Merriman writes also that he has another "good one" on Thompson, the first time he can leave the Wallow long enough to pay us a visit.

---

**The Department of Public Health.**—As announced in our Washington column last week, the Chairman of the Committee on the Department and Secretary of Public Health, Dr. Jerome Cochran, has appointed an auxiliary committee in Washington, consisting of Drs. H. L. E. Johnson, S. C. Busey, C. H. A. Kleinschmidt and J. R. Wellington. The Association bill has not been introduced this session, but the old Bureau bill has been introduced in the House. The Association does not ask for a Bureau; it wants a Department, and intends to be satisfied with nothing less.—*Journal A. M. A.*

**Charles Wood Bassett,** Secretary of the American Medical Publishers Association, has just issued a revised edition of the "Medical Journal Exchange List," containing the names and addresses of all publications in the United States and Canada, devoted to medicine, surgery, pharmacy, hygiene, microscopy, and allied science. This list is printed upon adhesive paper, and is used extensively by pulishers in mailing their exchanges, as well as by scientific writers in sending out reprints, etc. Price, $1.25 per dozen complete sheets. (Furnished free to members of the Association.)

---

**We** are requested by the Fort Worth Pharmacy Company, of Fort Worth, Texas, to say, that they will be pleased, upon application, to mail their Surgical Instrument Catalague and Price List to any member of the profession.

Their catalague quotes over six thousand articles and gives about the same number of cuts or figures of the articles.

They will also be pleased, at request, to mail special catalogues of Electric Batteries, Microscopes, Physician's Chairs and Tables and compressed air machines, which are now so successfully used by specialists in the treatment of throat and catarrhal diseases.

---

**The General Alumni Association** of the University of Pennsylvania was organized in June, 1895. Now, to further the object of the Association, and keep in touch with the *alumni* of the various departments,—and their name is legion, and they are scattered all over the earth,—the Association has begun the publication of a monthly organ, the *Alumni Register.* We have received the first number, January, 1896, and observe that Dr. Wm. Pepper's name is signed to the "greeting," but no name is given as editor. Dr. Ewing Jordan is Assistant Secretary of the Association, who will gladly give any information desired by interested parties.

---

**Orthopœdics.**—The JOURNAL is glad to learn that the Orthopœdic Hospital at Dallas, Texas, established last year by Dr. Edmondson, is succeeding splendidly. It is a demonstrated success; illustrating what intelligent enterprise, backed by professional ability and good judgment, will accomplish—even in the face of difficulties. It was thought by some of the older (and presumably wiser) members of the profession that such an establishment, outside of a large city, would be a dangerous experiment. Dr. Edmondson is to be congratulated, and Dallas felicitated on the inauguration of an establishment of this kind; it is quite a feature of the rising metropolis of Texas.

---

**Sulfonal in the Vomiting of Pregnancy.**—I have found this medicine a sure relief for this most obstinate trouble. I have been using it over a year, have recommended it to neighboring physicians, and never knew it to fail. Those who have tried it say it works like magic. It will relieve hiccough as effectually as well. My method of giving is 10 to 15 grains (60 to 1), dissolved in two tablespoonfuls boiling water, and given as hot as the patient can bear it. Sulfonal will stop vomiting of most any character, the quickest, safest and surest of any remedy in the category of medicine. I believe every physician should know this, and hope the *Journal* will pass it around.—*S. E. Burroughs, Allison, Iowa.*

---

**The Pennsylvania Colony Farm for Epileptics.**—The project for establishing a colony for epileptics, where country living and farm work, judiciously apportioned, would constitute the principal therapeutic treatment, which has been under consideration for some time, has at length assumed a definite shape. The court has granted a charter for the "Pennsylvania Colony Farm for Epileptics," and the corporators have organized, Novvember 13, by the election of Dr. Wharton Sinkler, president. Among the directors are Drs. Charles K. Mills. James C. Wilson, and Wharton Sinkler. A charitable genteman of Philadelphia has offered to give $50,000 for the erection of suitable buildings, provided the farm be secured before January 1, 1896.—*Boston Med. and Sur. Journal.*

---

**Extirpation of the Spleen.**—Schalita (*Arch. f. Kl., Chirurg.*, Bd. XLIX, H. 3) describes a case in a woman, 36 years of age, who has always been well, but in whom there had gradually developed a fluctuating tumor in the region of the spleen that filled the larger part of the left half of the abdomen. A laparotomy was made in the linea alba, and a large quantity of fluid of a brownish color was evacuated through a trocar. Splenectomy was then made, and recovery ensued. The tumor, which was as large as a man's head, was attached to the inferior surface of the spleen. This tumor or swelling is regarded as the result of hemorrhage either from the rupture of an aneurism of a branch of the splenic artery or from a subcapsular hemorrhage due to other causes, in consequence of which the cyst gradually formed. Examination of the blood for some time after the splenectomy did not show any changes in the corpuscles.—*Exchange.*

---

**The Latest Fad is Orchids.**—The blossoms are so lovely and so odd, and they grow anywhere, and are no trouble whatever, that anybody can have them. Most of them are *epiphytes;* that is, grow on trees, like mistletoe, and all you have to do is to tie the roots to a chip and hang them up where they will get sun and air. But heretofore the great cost has limited their possession to rich folks,—the New Jersey orchid dealers selling them at from $5 to $50 each. Hence, it will be a glad surprise to our readers to know that the *Mexican Plant Co.*, at Maravatio, Mexico, offer them to the JOURNAL readers at the ridiculously low price of 12 for a dollar, with 12 Mexican ferns thrown in. See their advertisement, and send and get some; it will repay you a hundred fold just to see an orchid bloom just once. Yours truly is growing them beautifully.

---

**Filled Cheese.**—A bill is now before congress, taxing, regulating the sale, importation, exportation, manufacturing and restricting the sale of filled cheese. Congressman Wilber, the author of the bill writes:

"It is a well known fact that the sale of filled cheese is resulting disasterously to the dairy interests of this country. The past year our exports have decreased over twenty-one per cent. on account of the bad repute which the American cheese has fallen into through the large shipments of the bogus product on the other side. Foreign consumers very soon became aware of the fact that filled cheese was being shipped from the United States to European markets, consequently became prejudiced against the American product, which resulted in the large decreased sales. Since filled cheese has been placed upon the markets, the exports have decreased from thirteen millions to seven millions annually. At the time the export sales amounted to the former figure it was largest amount received by European markets from any other country, and our product in quality and reputation was second to none.

The manufacture of filled cheese in Canada is prohibited by a stringent law, and English consumers being aware of that fact, and naturally prejudiced against any article produced in the United States, are very easily influenced to purchased the Canadian product.

We plead guilty to not knowing what "filled cheese" is; but if it is as bad as that, let's stop it.

**Medical Examiners' Fees.** — Our esteemed contemporary, *Puck*, has a very striking cartoon, *a propos* of the Holmes case, upon the subject of insurance companies and their employees. The point of the cartoon is that these companies are so eager to insure everybody and to make money that they are careless in their methods and secure cheap and incompetent men as their agents. The recent general cut in the prices for medical examiners' fees we have already spoken of, and stated that it was, we presumed, a purely business matter, and if they, the companies, could get good men for a lower price they had the right to do so. But, in the light of recent events, it seems that it is not likely they can get good men. Insurance companies had better try and economize in some other way than by cheapening the price paid to their agents for careful examinations of people that are to be insured.—*N. Y. Medical Record.*

[Some companies sponge service; get it for nothing. The New York Life refused to pay for information asked of us and furnished.—ED.]

---

**The Increase of Crime in America.**—The *New Orleans Times-Democrat* gives the following figures, showing the increase of murders and suicides in the United States in five years:

| | | | | |
|---|---|---|---|---|
| In 1890— | Murders, | 4,290 | Suicides, | 2,040 |
| 1891 | " | 5,906 | " | 3,331 |
| 1892 | " | 6,794 | " | 3,860 |
| 1893 | " | 7,615 | " | 4,436 |
| 1894 | " | 9,800 | " | 4,912 |
| 1895 | " | 10,500 | " | 5,759 |

The number of murders more than doubled in five years.

According to the United States census for 1850, there were in the United States a total of 6,339 prisoners; one in every 3341 of population. In 1890,—four decades,—the number had increased to over 82,000 prisoners, or one in every 759 of population. And these were prisoners; no estimate can be made, of course, of the number of criminals at large.

It would look as if there is something radically wrong with a system of dealing with crime and criminals under which such an alarming increase is possible. Is it any wonder that now and then the people take the law into their own hands, in a desperate hope of securing that protection which is not afforded by the law? The *Times Democrat*, commenting on the matter, says:

"Now is the time to overhaul our entire system of criminal laws; the laws themselves, the courts, and the juries. In this way only can we prevent those crimes which have become a scandal and a disgrace to the country."

It certainly does seem as if the time had come when a system so notoriously a failure should be abandoned, and some other plan tried. Not only a "scandal and disgrace," but dangerous to society, if not to the very foundation of government.

---

**Plymouth Rock Cockerels.**—Pure bred, of the famous Hefleybower prize strain. A few young cocks for sale. $2.50 each, cooped, on board. DR. DANIEL, Austin.

---

**Doctor,** show your wife the orchid advertisement of the Mexican Plant company; 12 for a dollar.

