# Erratum

**Published:** 1896-04

**Authors:** 


**Erratum.**—The JOURNAL was mistaken in saying that Prof. Geo. Dock, of the Michigan University Medical College, is an alumnus of Jefferson Medical College. He is from the University of Pennsylvania.

